# Preventing laboratory-acquired *Toxoplasma gondii* infection: literature review and case-based post-exposure prophylaxis proposal

**DOI:** 10.3389/fpubh.2025.1728709

**Published:** 2026-01-21

**Authors:** Diana Póvoas, Jocelyne Demengeot, Fernando Maltez

**Affiliations:** 1 Infectious Diseases Unit, Hospital de Curry Cabral, Unidade Local de Saúde São José, Lisbon, Portugal; 2 Lymphocyte Physiology, Gulbenkian Institute for Molecular Medicine, Oeiras, Portugal; 3 Instituto de Saúde Ambiental, Faculdade de Medicina, Universidade de Lisboa, Lisbon, Portugal

**Keywords:** accidental exposure, infection prevention, occupational exposure, post-exposure prophylaxis, *Toxoplasma gondii*

## Abstract

Occupationally acquired cases of the intracellular parasite *Toxoplasma gondii* have been reported, even though there is uncertainty on which infection prevention measures should be implemented in such event. We report a clinical case of laboratory-acquired toxoplasmosis and propose a protocol for post-exposure prophylaxis, given there are no formal guidelines. In this community case study, we describe the management and prevention protocol following an occupational laboratory exposure by a healthy 30-year-old *Toxoplasma*-seronegative female researcher who suffered an accidental needle puncture with a sample containing a genetically modified hypervirulent *Toxoplasma gondii* strain. Post-exposure prophylaxis (PEP) with trimethoprim-sulfamethoxazole was implemented for 4 weeks, during which seroconversion occurred, without any accompanying symptoms. *Toxoplasma* IgM and IgG positivity was observed 21 and 50 days after exposure, respectively, using a validated commercial electrochemiluminescence immunoassay (ECLIA, Roche). Follow-up was maintained for 1 year, during which the patient remained asymptomatic. This report highlights the importance of special care, surveillance and decision on the need for PEP upon occupational laboratory accidental exposure to *Toxoplasma gondii*. Since there are no guidelines on what the optimal PEP regimen should be, after a literature review, we propose a PEP occupational safety protocol to be implemented in laboratories that handle samples containing *Toxoplasma gondii*, either in clinical or research setting.

## 1 Introduction

*Toxoplasma gondii* is an infectious intracellular parasite that is usually transmitted through the ingestion of undercooked meat or other contaminated food (1). Data on *Toxoplasma gondii* seroprevalence exhibits significant regional and populational variation. A 2024 systematic review and meta-analysis that included 66 eligible studies, estimated a pooled global seroprevalence of 41% in workers with occupational animal exposure, the highest prevalence being found in Africa (51%) and the lowest in North America (23%; *p* < 0.05) (2). Another 2020 systematic review that included 250 studies found a global seroprevalence of 32.9% in pregnant women, the highest prevalence being found in the Americas (45.2, 95%CI: 33.4–53.4) and the lowest in the Western Pacific (11.2%, 7.8–15.1), with a statistically significant difference between regions (*p* < 0.0001) (3).

While the healthy immune system in humans usually develops protective immunity against the parasite without causing disease, individuals with weakened immune systems and pregnant women can experience severe cases of toxoplasmosis. Laboratory work with *Toxoplasma gondii* usually requires biosafety level 2 (BSL2), a classification reserved for “not life-threatening pathogens for which therapies exist” and consisting of basic restrictive environment (4). BSL-2 classification requires personal protective equipment (PPE) with gloves and eye protection to be worn at all times. All procedures that may produce aerosols or involve high concentrations or large volumes should be conducted in a biological safety cabinet. BSL-2 classification is applicable to infectious agents associated with human disease, not highly transmissible, treatable, not airborne, such as *Salmonella* spp., *Brucella* spp., *Listeria monocytogenes* (4). In the laboratory setting, contamination with infective *T. gondii* parasites can occur through accidental direct injection into the skin, blood or lymph, or through contact with mucosal surfaces such as the mouth or nose, or splashes into the eye. *Toxoplasma gondii* strains used in research are often genetically modified, which may associate with different risk profiles. Occupationally acquired cases of *Toxoplasma gondii* infection by different transmission routes in the laboratory setting have been reported, from asymptomatic to severe illness (1). By 2001, 47 laboratory-acquired cases of *Toxoplasma* infection had been reported, including four cases of encephalitis, two of whom also developed myocarditis, one of which died (1). Despite the recognition of occupational exposure risk and reported cases of laboratory-acquired *Toxoplasma* infections, there are no formal guidelines for occupational post-exposure. To address this gap, we report the clinical management of an occupational exposure to a genetically modified hypervirulent *Toxoplasma gondii* strain and, after literature review, propose a structured post-prophylaxis protocol.

## 2 Context

A healthy 30-year-old female researcher working in a biosafety level 2 laboratory, in April 2024 suffered an accidental needle puncture from a syringe used to administrate a genetically modified hypervirulent *Toxoplasma gondii* strain to experimental animals. The strain involved was the genetically modified *T. gondii* RHΔHXGPRT, a Type I lineage. The inoculum consisted of RHΔHXGPRT tachyzoites propagated *in vitro* in human foreskin fibroblasts, the standard method for maintaining this life stage in experimental models (5). This strain lacks the hypoxanthine-xanthine-guanine phosphoribosyltransferase (HXGPRT) gene, which is commonly used as a selectable marker for stable genetic transformation in *T. gondii* research (6). Prior to administration, these parasites were released from host cells by repeated syringe lysis, which mechanically disrupts the parasitophorous vacuole membrane. This technique, while standard in *T. gondii* research, inherently involves manipulation of infectious material using needles under pressure, thereby increasing the risk of accidental inoculation through needle-stick injuries—even in the presence of full BSL-2 protective measures (5). Despite wearing appropriate PPE, the laboratorian perforated a glove with the syringe needle, resulting in visible bleeding from a puncture wound. This illustrates how routine syringe-based manipulation of high-concentration infectious material may increase the risk of accidental inoculation, even when standard BSL-2 precautions are followed.

The wound was cleaned and medical advice was immediately sought, according to laboratory protocol. After an initial observation in the emergency department on the same day, the patient was referred to our infectious diseases practice. Upon observation 3 days after exposure, she was asymptomatic, reported no relevant personal history or drug allergies, and the observation was unremarkable. Baseline laboratory tests included a complete blood count, liver and renal function panels, C-reactive protein, and a pregnancy test. Anti-*Toxoplasma* IgG and IgM were assessed at baseline and during follow-up using commercial ECLIA kits. Laboratory data showed normal blood counts, liver and kidney parameters, and negative C-reactive protein level. The pregnancy test result was negative. Although the ΔHXGPRT mutation is not directly linked to increased virulence, the parental RH strain is acutely virulent in mice, and the genetic manipulation does not attenuate its infectivity or replication capacity. Given this, and the small inoculum needed to cause infection in the laboratory setting, the exposure was considered high-risk (1).

## 3 Details to understand key programmatic elements

### 3.1 Case management

Given the needle puncture from a syringe containing a concentrated solution of *Toxoplasma* parasites, which penetrated both the glove and skin causing minor bleeding, we considered the risk of transmission to be high. This risk assumption, together with published cases of serious infection in this context, were the rationale for post-exposure prophylaxis (PEP) to be started. However, there are no published recommendations for drug regimens in this context, apart from some published institutional laboratories internal procedures that, amid possible – but not consensual – pharmacological prophylaxis strategies to be considered, conclude that medical evaluation should always be sought (7, 8).

Uncertainties regarding risk assessment were discussed with the exposed laboratorian, along with available drug regimens and possible toxicity concerns, after which a shared decision was reached to start PEP with trimethoprim-sulfamethoxazole (TMP-SMX), 5 mg/kg of trimethoprim component twice daily. While pyrimethamine-based regimens are commonly used for toxoplasmosis treatment in immunocompromised individuals, preference for prophylaxis with TMP-SMX was given, due to availability, favourable safety profile, and published evidence on its efficacy in treating acute *Toxoplasma* infections (9, 10). PEP duration was determined to last for two or 4 weeks, according to clinical and serology evolution. On day 12 of PEP – 21 days after accidental needle puncture, anti-*Toxoplasma* IgM was positive.

**Figure 1:**
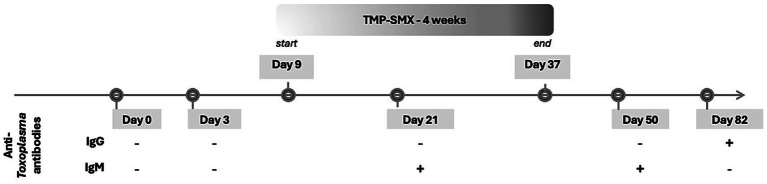
Clinical case timeline of events. TMP-SMX, Trimethoprim-sulfamethoxazole.

Seroconversion was again confirmed with IgM positivity and later IgG positivity and IgM negativity on day 50 and day 82 after accidental exposure, respectively. Anti-*Toxoplasma* IgM and IgG antibodies were assessed using a validated commercial electrochemiluminescence immunoassay (ECLIA; Roche Diagnostics) at baseline and during follow-up. The patient remained asymptomatic throughout this period and remains symptom-free 1 year after. Timeline of events is represented in Figure 1.

### 3.2 Protocol proposal

While there are no guidelines on the need to start *Toxoplasma* PEP after accidental exposure and, should that be the case, which drug or drug combination should be used, some laboratory safety procedures admit such strategy to be implemented, upon medical observation (7, 11). After literature review, we suggest the following post-exposure protocol to be implemented in laboratories that handle samples containing *Toxoplasma gondii*, either in the clinical or research setting (Table 1). We admit similar treatment regimens to the ones used for treatment of acute infection in immunocompetent individuals may be considered effective in the setting of laboratory occupational exposure.

**Table 1:** Recommended post-exposure prevention regimens.

Regimen	Adult dosing and daily pill burden	Side effects	Duration
Preferred			
Trimethoprim-sulfamethoxazole	5 mg/kg trimethoprim, orally twice daily 4 pills/day	Rash, fever, leukopenia, hepatitis, nausea, vomiting, diarrhoea, crystalluria; rarely, Stevens-Johnson syndrome	2 to 4 weeks*
Alternative			
Pyrimethamine + sulfadiazine + leucovorin	Pyrimethamine 100 mg loading dose, followed by 25–50 mg daily Sulfadiazine 2–4 g daily in 4 divided doses Leucovorin calcium 10–25 mg daily 7–14 pills on day 1, then 6–12 pills/day	Rash, nausea, and leukopenia	
Pyrimethamine + clindamycin + leucovorin	Pyrimethamine 100 mg loading dose, followed by 25–50 mg daily Clindamycin 300 mg four times daily Leucovorin calcium 10–25 mg daily 7–10 pills on day 1, then 6–8 pills/day	Fever, rash, and nausea; diarrhoea due to risk of production of *Clostridioides difficile* toxin	

* Duration may be guided by anti-*Toxoplasma* antibody assessment at 2 weeks. A total 4-week duration is recommended if antibodies anti-*Toxoplasma* are detected at 2 weeks.

Upon accidental exposure in the lab, personal protection equipment should be immediately removed, the exposed region should be thoroughly washed with running water and medical care should be sought to assess seropositivity for *Toxoplasma gondii*. If seropositivity is not confirmed prior to the event, both anti-*Toxoplasma* IgG and IgM should be assessed.

When evaluating PEP need, risk of laboratory-acquired *Toxoplasma* infection may be difficult to be precisely ascertained, since this is likely influenced by inoculum size, strain virulence, host immune status and route of exposure. Insights into *Toxoplasma* transmission, life cycle and pathogenicity have shown that, upon common exposure routes such as faecal-oral or vertical transmission, the speed on which parasites can disseminate and establish cysts in tissues, is influenced by the strain virulence and the host’s immune system and an inoculum as small as 10 oocysts is considered to be infectious (7, 12). In the absence of a clearly defined time window for these stages to take place after exposure, we suggest that PEP should be started as soon as possible, ideally in the first 72 h after exposure. However, in our expert opinion it may be reasonable, in high-risk exposure settings, such as high inoculum or hypervirulent strains, that this strategy should be considered up until 7 days after exposure, since described median incubation periods of laboratory-acquired infections are up to 8 days (range 3–60 days), comparable between parenteral and mucosal exposures (1).

Anti-*Toxoplasma* IgG and IgM should be reassessed 2 weeks after prophylaxis is started, since this is often the time period seroconversion was detected in published laboratory-acquired Toxoplasma infection episodes (1). If negative and no clinical symptoms have developed, stopping prophylaxis may be considered. If either IgM or IgG are positive, our expert opinion is that total treatment duration should be extended to 4 weeks. In case of previous or baseline anti-*Toxoplasma* IgG positivity, there is no evidence to support the need for PEP to be implemented.

Laboratorians with accidental exposure to *Toxoplasma* should be monitored for clinical and laboratory evidence of infection (1). Additional exams may be performed, depending on clinical and laboratory data, specifically if acute, systemic or complicated infection is suspected. While TMP-SMX is generally well-tolerated, sometimes gastrointestinal intolerance, rash, and, less frequently, renal or haematological toxicity may ensue. Therefore, monitoring of complete blood count along with renal parameters should be warranted, particularly in prolonged courses or in individuals with pre-existing comorbidities.

## 4 Discussion

This case highlights the importance for special care, surveillance and decision on the pertinence for PEP upon occupational accidental exposure to *Toxoplasma gondii* in the laboratory, since this setting may represent a rather unnatural route of transmission, with often genetically modified parasites and higher inoculums of parasites. In line with authors Herwaldt (1) and Sini (13), we too advocate that all *T. gondii* isolates should be considered pathogenic for humans, even if they are avirulent for mice.

Our report describes a high-risk exposure to this microorganism: parenteric inoculation through a needle-stick injury with a high inoculum of a genetically modified hypervirulent *Toxoplasma* strain.

Because *T. gondii* can cause systemic infection detectable by serologic testing, we recommend that personnel who routinely handle *Toxoplasma*-containing infectious samples undergo baseline IgG testing at the time of employment (1). Although not systematically implemented in all laboratories, as is the case described, such screening may help guide risk assessment and post-exposure decision-making.

In cases where a laboratorian with negative anti-*Toxoplasma* IgG antibodies ensues accidental exposure to *Toxoplasma*-containing samples, PEP may be warranted. While there is no published evidence on the efficacy of such a strategy, since available evidence suggests infection risk to be significant, as well as risk of moderate to severe infection, need for PEP must be evaluated.

A 2001 review that accounted for 47 cases of laboratory-acquired *Toxoplasma* infections, reported between 1924 and 1999 in six languages, included one reported death (1). A more recent review on cases that included 164 published reports from 2000 to 2021, accounted for three occupational cases of *Toxoplasma* infection, worldwide (14). In the former study, reported cases of laboratory occupational exposure to *Toxoplasma* estimated one episode per 24 person-years rate of infection (1). In this same review of 47 episodes laboratory-acquired infection with *Toxoplasma gondii*, exposure routes were parenteral (29.8%), presumptive ingestion (19.1%), or due to mucous membrane exposure (17%). In a significant proportion or the reported cases, there was no recognition of the moment accidental exposure took place. We believe the declining trend in the number of laboratory-acquired *Toxoplasma* infection since the 1950 shown in both reviews by Herwaldt et al. (1) and Blacksell et al. (14), most likely is the result of improvement in laboratory biosafety recommendations, rather than progression in occupational health procedures regarding occupational exposure to *Toxoplasma*, since most of international biosafety guidelines do not contain specific guidance regarding this accidental exposure in the laboratory setting (4, 11).

In our report, given serological markers confirming seroconversion upon exposure to a hypervirulent strain and uncertainty regarding the natural course of disease in humans, even if immunocompetent, TMP-SMX was maintained for an additional 2 weeks, totalling 4 weeks. This is the usual approach for individuals with acute infection and treatment criteria, such as pregnancy, underlying condition that may be a risk factor for severe disease such as immunocompromise, or genotype virulence (15, 16). In these cases, treatment of *Toxoplasma* acute-infection is usually performed with a two to four-week course of combination of drugs. The recommended regimens include pyrimethamine plus sulfadiazine plus leucovorin or, pyrimethamine plus clindamycin plus leucovorin.

Treatment with TMP-SMX may also be used for a course of 2 to 4 weeks. Furthermore, in immunocompromised individuals such as people living with HIV and transplant recipients, this drug combination may also be used for primary prophylaxis or secondary prophylaxis to prevent relapse after acute infection treatment (9, 10, 17). Several publications have shown TMP-SMX has a favourable safety profile and non-inferior efficacy comparing to other drug regimens (9, 18–20). Pyrimethamine-based regimens may have limited availability in some settings and are associated with bone marrow suppression, dermatologic, and gastrointestinal side effects (21). Considering availability, potential side-effects, treatment efficacy, pill burden, drug availability and the need for analytical and clinical monitoring during treatment, it is our expert opinion that preference should be given to TMP-SMX post-exposure prophylactic regimen, if there is no evidence of sulphonamide allergy. TMP-SMX should be started, with a dosage based on the trimethoprim component (5 mg/kg of trimethoprim component, orally twice daily). In case of sulphonamide allergy, an alternative treatment regimen should be initiated, as described in Table 1, considering potential side-effects, drug availability and pill burden.

To reduce the risk of similar incidents, we recommend for enhanced sharps handling protocols to be implemented, refresher training on *Toxoplasma*-specific biosafety risks, and baseline serological screening for laboratory staff. Institutions should also consider incorporating *Toxoplasma*-specific PEP guidance into their biosafety manuals, ensuring that exposed individuals receive rapid risk assessment and appropriate prophylaxis when indicated.

We believe this case report together with the literature review performed address the need for formal guidance and PEP protocols to be implemented in institutions that handle *Toxoplasma*-containing samples, either in the research or clinical setting. Moreover, this work highlights the knowledge gaps in the natural course of laboratory-acquired *Toxoplasma* infection, PEP effectiveness and the lack of long-term follow-up data. Development of systematic data collection, either by multicentre studies or national/international registries, would support evidence-based protocols and clinical management strategies, contributing to improvement of occupational health procedures.

## Data Availability

The raw data supporting the conclusions of this article will be made available by the authors, without undue reservation.
